# The origin of chert in the Aurignacian of Vogelherd Cave investigated by infrared spectroscopy

**DOI:** 10.1371/journal.pone.0272988

**Published:** 2022-08-17

**Authors:** Benjamin Schürch, Stefan Wettengl, Simon Fröhle, Nicholas Conard, Patrick Schmidt

**Affiliations:** 1 Department of Early Prehistory and Quaternary Ecology, Institute of Prehistory, Early History and Medieval Archeology, University of Tübingen, Tübingen, Germany; 2 Senckenberg Centre for Human Evolution and Paleoenvironment, University of Tübingen, Tubingen, Germany; 3 Applied Mineralogy, Department of Geosciences, Eberhard Karls University of Tübingen, Tübingen, Germany; Sapienza University of Rome: Universita degli Studi di Roma La Sapienza, ITALY

## Abstract

The analyses of raw material provenance offers the possibility of tracing short and long-distance raw material transport. So far, most studies of raw material of flint and chert in Europe have been based on macroscopic analyses. We apply infrared spectroscopy to Aurignacian assemblages from Vogelherd cave and to the Magdalenian site Randecker Maar in southwestern Germany. We compare raw material samples from three chert-bearing areas in Germany with archaeological samples from Vogelherd. Our results show that infrared spectroscopy can distinguish between different raw materials. Our archaeological samples from Vogelherd correspond to the sampled geological cherts in terms of their spectral signature. Our comparison of reference samples and archaeological samples highlights problems in commonly used macroscopic identifications of chert raw materials.

## Introduction

Archaeological raw material studies have the potential to provide information about territoriality or settlement dynamics [e.g. 1–5] among many other human behavioural traits. These questions are of major importance in the framework of the European early Upper Palaeolithic when important population turnovers took place. The Upper Palaeolithic Vogelherd Cave, with its early figurative art, musical instruments and personal ornaments, is one of the major sites for our understanding of early anatomically modern humans in Central Europe [6–8]. The site is situated in the Swabian Jura region, a limestone plateau oriented southwest to northeast in southern Germany. The Jurassic deposits in the vicinity of the site are rich in chert (Jurassic chert) and different varieties of Tertiary chert can be found on the northern side of the Jura. Several studies of raw materials have been conducted on Middle and Upper Palaeolithic sites of the region [9–20]. Another site that we consider in this study, and having yielded Magdalenian artefacts, is the Randecker Maar, an open-air site located on the southern rim of the crater also named Randecker Maar as well [21].

The identification of siliceous raw materials in southern Germany has so far almost exclusively been conducted through macroscopic inspection. Such macroscopic analyses are based on comparisons between samples using low magnification lenses or stereomicroscopes [22]. Features like colour, lustre and translucency are commonly used for identifying chert materials [4, 10, 22–28]. However, macroscopic analyses are not always reproducible and may be prone to observer bias. In this study, we use infrared (IR) spectroscopy, a method proposed by Parish [5], to verify some of the hypotheses on the origin of chert raw materials from Vogelherd that were previously identified based on macroscopic analyses [9, 21, 29]. Parish’s method is based on non-destructive measurements of the IR reflectance [see for example 30–36] on the surface of artefacts, using commercially available reflection spectrometers. The so-obtained spectra express several parameters, such as crystal lattice bonding parameters, mineralogical content, and crystallographic properties, of the analysed samples. The spectra can therefore be used to establish similarities and dissimilarities between different groups of samples. We compare a selection of artefacts from Vogelherd cave and Randecker Maar that have previously been determined macroscopically with three raw material outcrops, using this method.

## Materials and methods

### Archaeological sites

We selected artefacts from Vogelherd and in a second step from the Magdalenian site, Randecker Maar. The artefacts from Vogelherd constitute the main corpus of this study. The artefacts from the Randecker Maar site allowed us to verify whether the spectroscopic analysis was able to distinguish between artefacts from different sites and to determine whether surface phenomena might influence the measurements. If artefacts from different sites yielded similar signals, but raw material samples from different outcrops didn’t, we could exclude that taphonomic alterations influence our analysis. If, however, artefacts from different sites can be distinguished, it may be possible to correlate them with distinct reference samples.

Vogelherd Cave near Niederstotzingen is located in the Lone Valley of the Swabian Jura in the district of Heidenheim. The site was completely excavated in 1931 by Tübingen-based archaeologist Gustav Riek [20], who defined nine archaeological horizons. Of these, layers IV and V are of Aurignacian age [20], as determined by numerous radiocarbon dates [6]. Between 2005 and 2012, excavations were carried out in the backdirt of Gustav Reek’s initial excavation under the direction of one of us [N.J. Conard, see: 7]. This excavation revealed numerous artefacts that had been overlooked during the excavation in 1931. For this study, we use Aurignacian artefacts from layers IV and V from the original excavations and samples from the new excavation, we assigned to the Aurignacian based on technological and typological analysis. These artefacts (N = 19) include tools such as pointed blades, end-scrapers and burins. Besides the tools, blanks and cores were also included.

As mentioned above, we also included artefacts from the Magdalenian open-air site of Randecker Maar, to compare our results to the Tertiary chert from Vogelherd cave. The reason for choosing this site is its geographic proximity to the Randecker Maar, the purposed raw material source of one of the chert varieties from Vogelherd. Another reason why the Magdalenian of the Randecker Maar was chosen for comparison is that there are no Aurignacian sites in the vicinity of the Randecker Maar. The site is located on the southern rim of the ~1.2 km wide Randecker Maar volcanic crater and is situated approximately 50 km to the west of Vogelherd [21]. The lithic artefacts from Randecker Maar come from surface assemblages collected by G. Romberg [21]. The dominant raw material is described to be Tertiary chert from within the Randecker Maar crater [21, 37]. For the Randecker Maar, tools, cores and blanks were also included in this study (N = 25). A detailed description of the assemblage from Randecker Maar was presented by Wettengl [21].

### Geological setting and raw material outcrops

**Fig 1:**
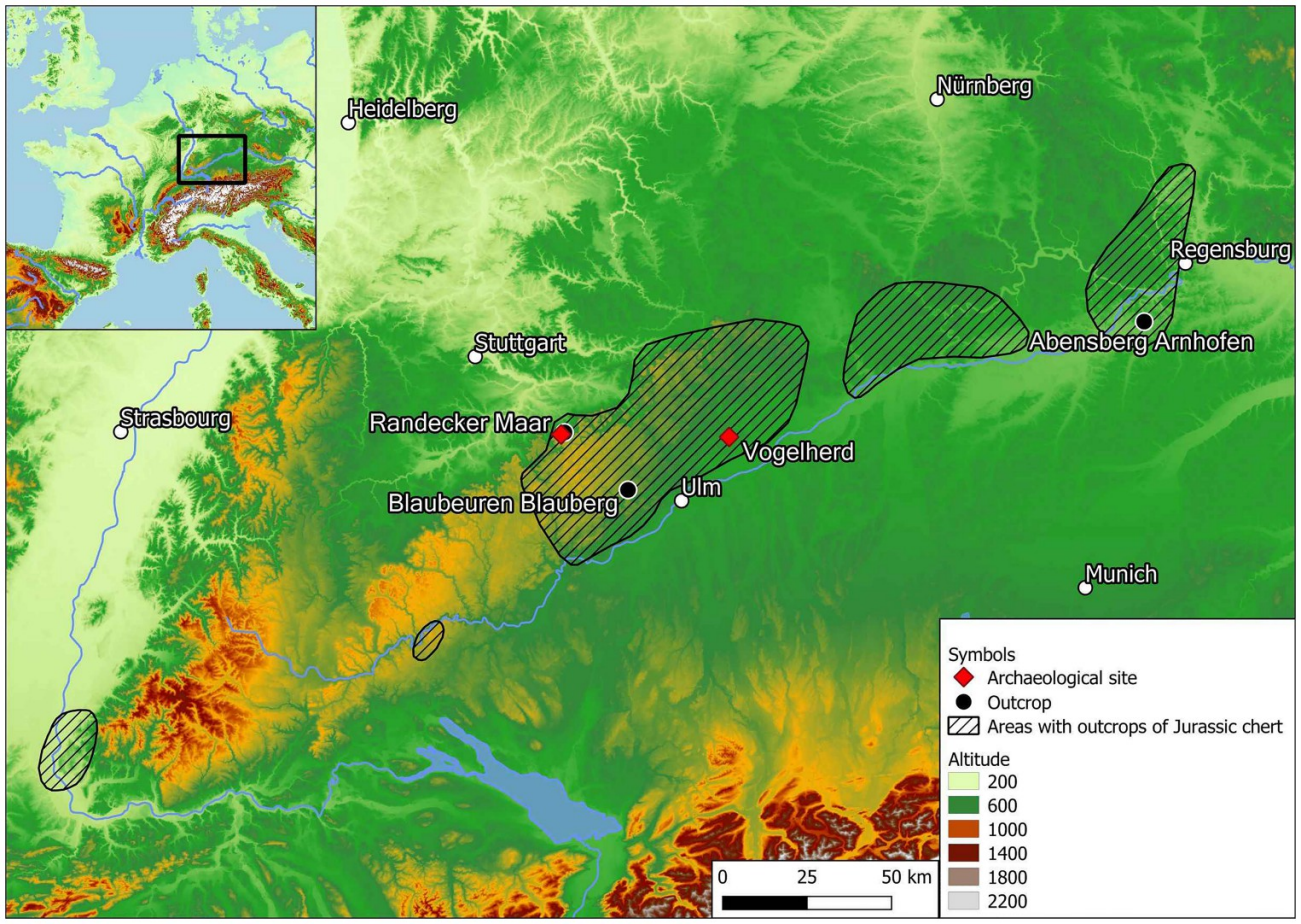
Map of Southern Germany with the included archaeological sites, outcrops of Jurassic and Tertiary chert. Basemap: © European Union, Copernicus Land Monitoring Service 2020, European Environment Agency (EEA)", f.ex. in 2018: “© European Union, Copernicus Land Monitoring Service 2018, European Environment Agency (EEA)”, with funding by the European Union.

**Fig 2:**
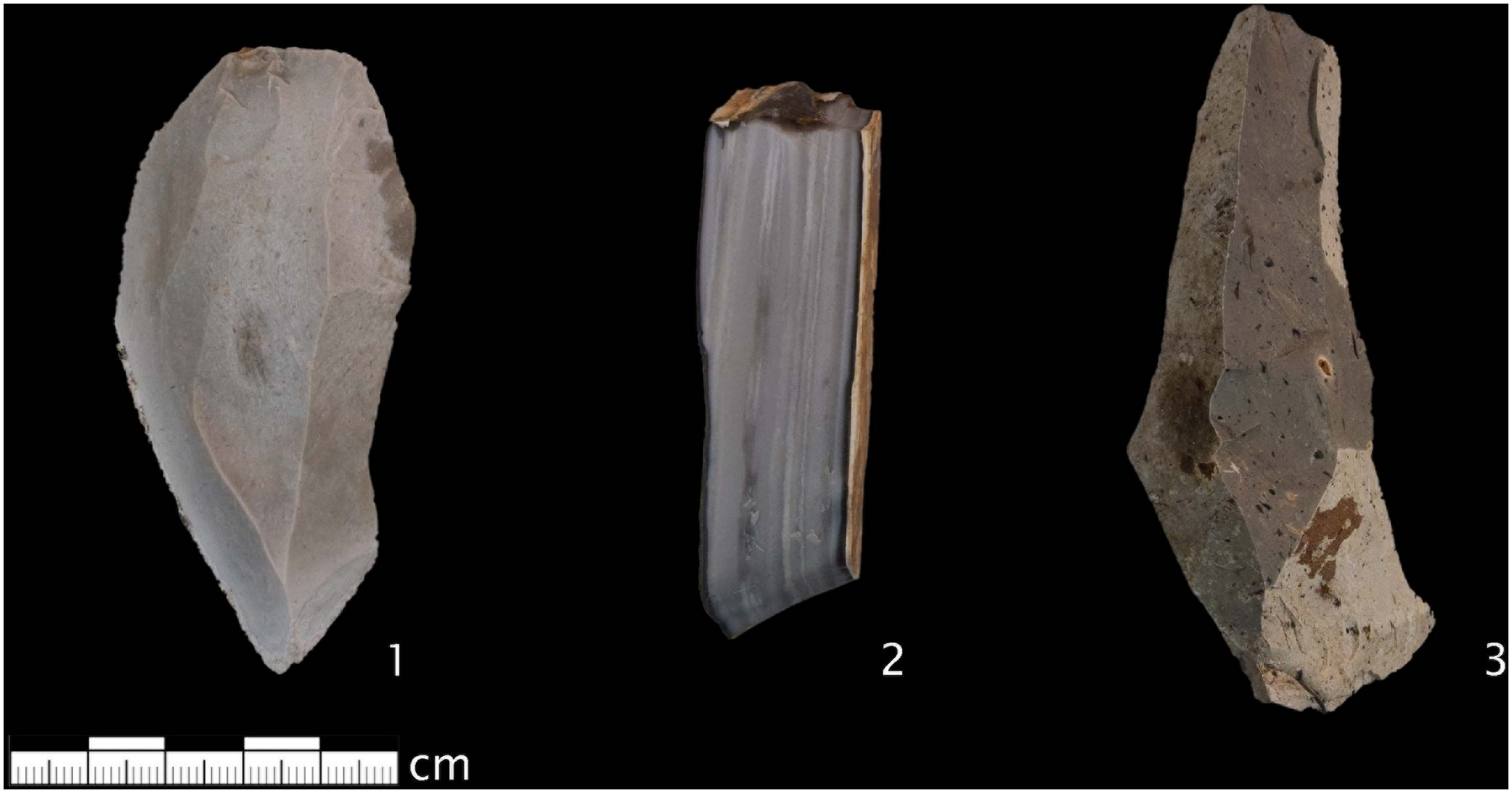
Chert from the three outcrops considered in this study. 1. Blaubeuren Blauberg, 2. Abensberg Arnhofen, 3. Randecker Maar. Photos taken by B. Schürch.

The Swabian Jura, as one of the central geological formations in southern Germany located north of the Danube, which forms one of its natural borders (Fig 1). Raw materials such as radiolarite, hydrothermal quartz and quartzite can be found in the Danube and its tributaries. To the west, the Black Forest separates the Swabian Jura from the Swiss and French Jura. To the north of the Swabian Jura, locally available chert raw materials include Muschelkalk chert, Triassic chert and Keuper chert (Fig 1). Jurassic chert from Blaubeuren Blauberg, Bavarian tabular Jurassic chert from Abensberg Arnhofen and Tertiary chert from the Randecker Maar, that were formed in a former tertiary lake in the crater of an extinct volcano, are the three raw material deposits that were included in the study (Fig 2). The selection of geological samples made here may not show the full variability of the chert types.

All samples used in this study come from secondary deposits in the Swabian Jura region. Deposits of primary raw material are rarely found in the Swabian Jura region and today occur almost exclusively in modern quarries. The raw material nodules and slabs have weathered out of their primary layers and are found in the overlying clays [12]. The raw material deposits we have selected for this study are well documented, and some of them are described in previous studies [10, 21, 26, 38, 39]. The *Blaubeuren Blauberg* outcrop (S1 Fig) is located above Blaubeuren in the vicinity of the caves of the Ach- and Blautal and is approximately 30 km from Vogelherd. The outcrop in which chert can be found today result from the weathering of a Malm Zeta 1 horizon of the Jurassic [10]. This raw material deposit is surrounded by Palaeolithic and Neolithic sites as well as other raw material outcrops [14, 39–41]. The secondary deposits from near the town of Blaubeuren were mined in the Neolithic period [40, 42]. The raw material from *Blaubeuren Blauberg* occurs in nodules and is usually banded concentrically. The colour palette of this material ranges from light to dark grey.

The *Abensberg Arnhofen* raw material outcrop (S2 Fig) is known to have been mined intensively during the Neolithic period [38, 43, 44], although this chert was also used during the Palaeolithic [19, 29, 45]. Chert from *Abensberg Arnhofen* differs from chert from *Blaubeuren Blauberg* mainly in its platy appearance and banding. This tabular chert can also occurs in round aggregates [38]. The reason for the plate-like morphology is that it formed in shallow water bays [38]. The horizon from which this chert can be collected derives from the Malm Zeta 1 and 2 strata of the Jurassic [38]. Tabular cherts from *Abensberg Arnhofen* are characterized by their cortex parallel banding and are usually between 0.5–4 cm thick [38]. The round nodular chert variants from Abensberg also show banding that runs concentrically [38]. Nodules have an average diameter of 10–20 cm [38]. The colours within this raw material range from light gray to dark gray, which partially fades to a dark blue color.

The Randecker Maar, which is located on the north-western rim of the Swabian Jura, is a geologic landmark and a well-known raw material outcrop of Tertiary chert (S3 Fig). It belongs to the group of volcanos between Bad Urach and Kirchheim unter Teck that consist of approximately 350 vents. Tertiary chert formed in this volcanic lake (Maar in German) and can be found there in secondary outcrops [37, 46, 47]. Auffermann [37] claims that the Randecker Maar is the only well-known outcrop of Tertiary Chert.

All raw material used for this study were housed in the raw material reference collection of the Department of Early Prehistory at Tübingen University (Table 1). These samples were collected by three of us (SW, BS, SF) and by colleagues W. Burkert, H. Floss, M. Kaiser and M. Siegeris.

**Table 1:** Geological samples analysed.

Raw material outcrop	Number of Blocks	Number of spectra
Blaubeuren Blauberg	10	137
Abensberg Arnhofen	9	155
Randecker Maar	6	110
Total	25	402

To obtain data that reflect the varieties of the deposits, we used at least six raw material nodules or plates from each raw material outcrop. These aggregates were then broken up into smaller pieces so that multiple sub-samples could be analysed. We used the raw material nodules/plates from each outcrop to produce blades and flakes for subsequent analysis.

### Infrared spectroscopic analysis

Mid-IR reflectance was recorded between 1250 and 600 cm^-1^ with a portable Agilent Cary 630 FTIR spectrometer (using unpolarized light and a 10° angle of incidence). Following Parish [5], spectra were collected on fracture surfaces, and besides the knapping to expose the fracture surfaces no sample preparation was necessary. Spectral acquisition was repeated and averaged 160 times and the resolution was set to 8 cm^-1^ on the instrument. These reflectance spectra display quartz lattice bands that can be assigned to several stretching modes [48, 49] of the chert’s quartz crystals. Band shape is influenced by optical phenomena occurring in these crystals (for an example see: [50]).

Spectra were further treated by calculating their first derivative (after 17-point smoothing), which was plotted over wavenumber. We then performed a principal component analysis (PCA) on the first-derivative data, reducing the relatively high dimensionality of the data to 2 [51]. This complexity reduction of the spectral data mitigates the effect of overfitting when calibrating classifiers [52]. The resulting PCA plots display the variance between different samples in terms of the shape of IR absorption bands (Fig 5). This promises to yield information on differences in band parameters and shapes which in turn, is expected to reflect crystallographic factors, such as weak OH-bands and other hydrogen-related impurities [53, but see also: 54]. Similarities and dissimilarities between samples, as observed from the variance in PCA plots, can therefore be related to the sample’s mineralogical composition and variations in quartz crystallography In a second step we conducted linear discriminant analysis (LDA) with our archaeological samples included but without assigning them to groups [49]. To validate our dataset of the LDA, we performed accuracy assessment test on the three raw material groups. Archaeological samples were not included in this step. For this, a random sample set of 10%, 20% and 30% was removed from the groups. These random subsamples were then assigned by the LDA to the three groups to see if they would be assigned correctly.

## Results

### Macroscopic determination

**Fig 3:**
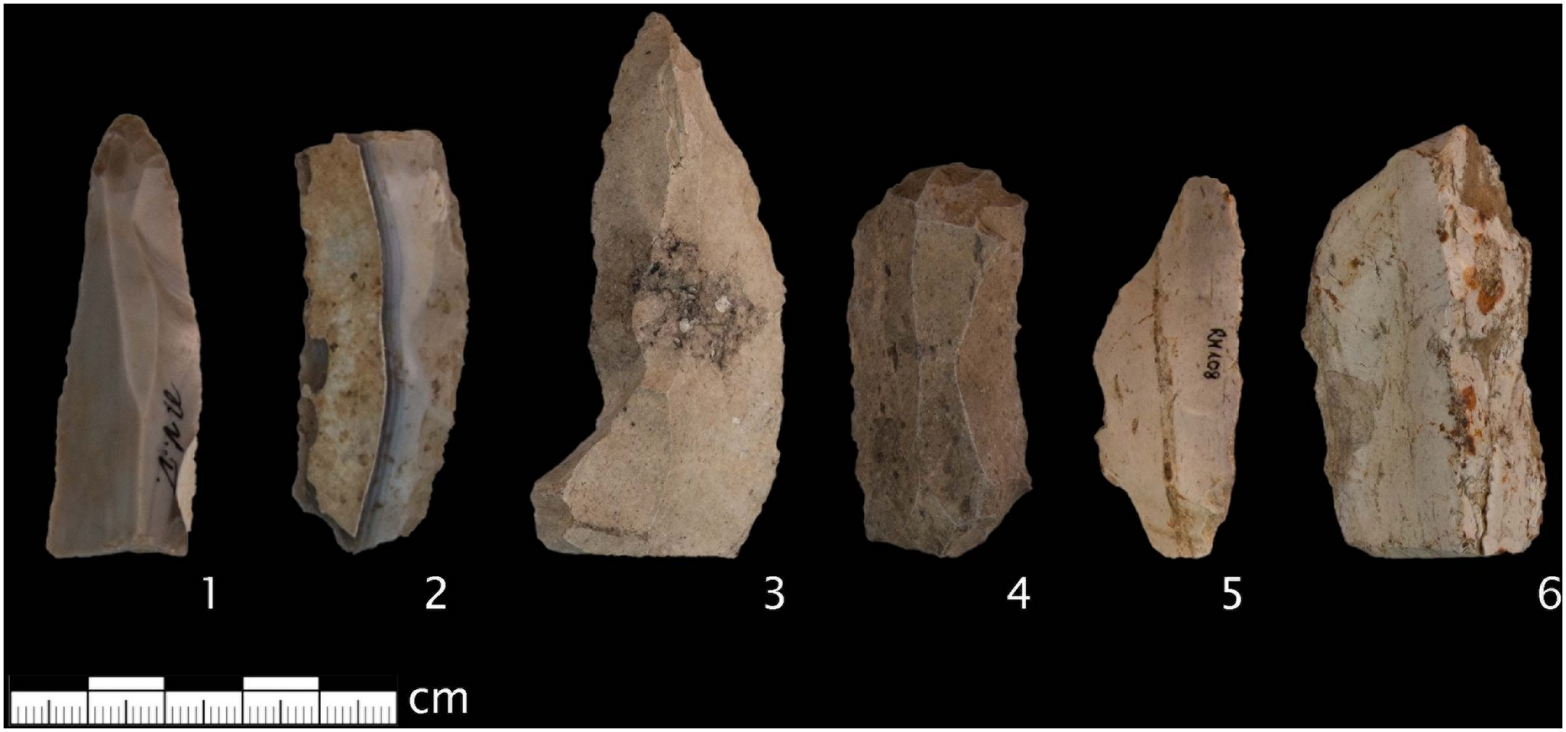
Archaeological samples. 1, 2 Jurassic cherts from Vogelherd, 3, 4 Tertiary cherts from Vogelherd, 5, 6 Tertiary cherts from Randecker Maar. Classifications based on macroscopic criteria. Photos by B. Schürch.

Based on macroscopic characteristics raw material samples from Blaubeuren Blauberg, Abensberg Arnhofen and Randecker Maar differ from each other. The main criteria we used for the macroscopic determinations were colour, lustre, translucency. A portion of the artefacts from the Vogelherd (N = 9) and all artefacts from the archaeological site Randecker Maar (N = 25) showed macroscopic characteristics similar to Tertiary chert from the Randecker Maar (N = 34) (Fig 3 (3–6); Table 3). The remaining samples from Vogelherd (N = 10) were macroscopically determined to be Jurassic chert (Fig 3 (1, 2)). Most of them show cortex, parallel banding, and the same colour palette as raw material samples from Abensberg Arnhofen and Blaubeuren Blauberg.

### Infrared spectroscopy

#### Geological samples—PCA results

**Fig 4:**
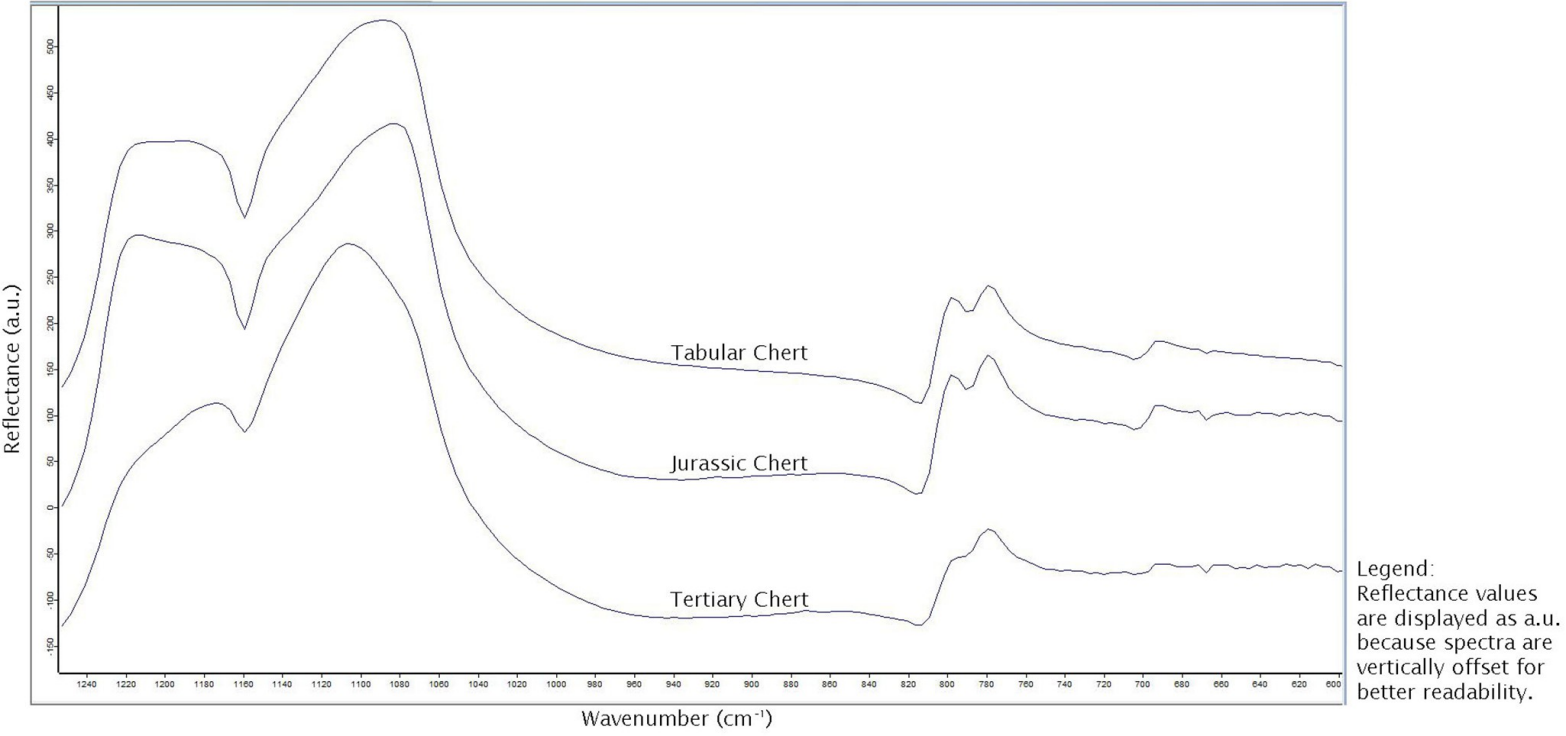
Example spectra of the three raw material outcrops tested.

**Fig 5:**
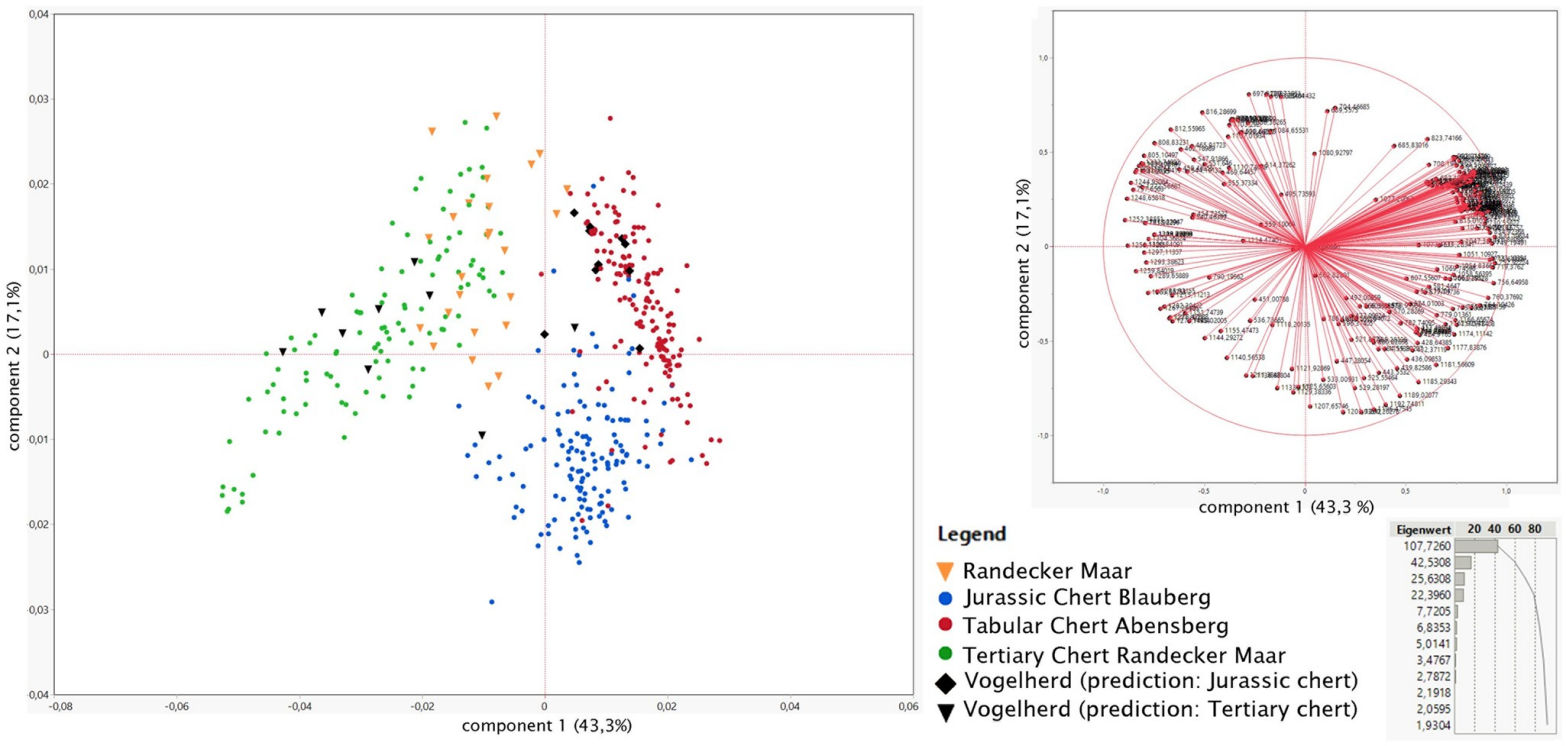
PCA scatter plot (covariance matrix) resulting from the first derivative data of these spectra (left) and the loading plot showing the direction different variables (in our case different wave numbers) plot in the PCA biplot and first two principal components (top right); and the eigenvalue (right bottom). All archaeological samples from the Randecker Maar were macroscopically predicted to be Tertiary chert, these are shown as orange triangles. The samples from Vogelherd predicted to be Tertiary chert by macroscopical analysis are shown as black triangles. The samples predicted to be Jurassic chert from Vogelherd are shown as black diamonds.

Fig 4 shows an example of each of the raw material outcrop. The spectrum shown was used in its entirety for PCA. Fig 5 shows the PCA plot created on the base of the infrared reflectance spectra of three of the samples in the study region. The PCA plot (Fig 5) shows clear separation of all three raw material groups. First-derivative spectra of Jurassic chert reference samples from Blauberg and Arnhofen are separated from Tertiary chert coming from the Randecker Maar. The former two overlap, but plot over a large area that allows separating both of the clusters.

#### Archaeological samples—PCA results

Most of the archaeological samples from the Randecker Maar (N = 21) plot in the same region as the Tertiary chert. Only few samples (N = 4) from the Randecker Maar plot near the Jurassic chert scatter, however they do not plot directly onto Jurassic chert. The Vogelherd archaeological samples predicted to be Jurassic chert by our macroscopical analysis (N = 10) all plot onto the geological reference of the Jurassic chert. Archaeological Vogelherd samples predicted to be Tertiary chert by macroscopical analysis (N = 7) mostly plot onto the geological reference of Tertiary chert. The exceptions to this are two artefacts that plot at the edge of the Jurassic chert from Blaubeuren Blauberg.

#### Linear discriminant analysis (LDA)—Results

**Fig 6:**
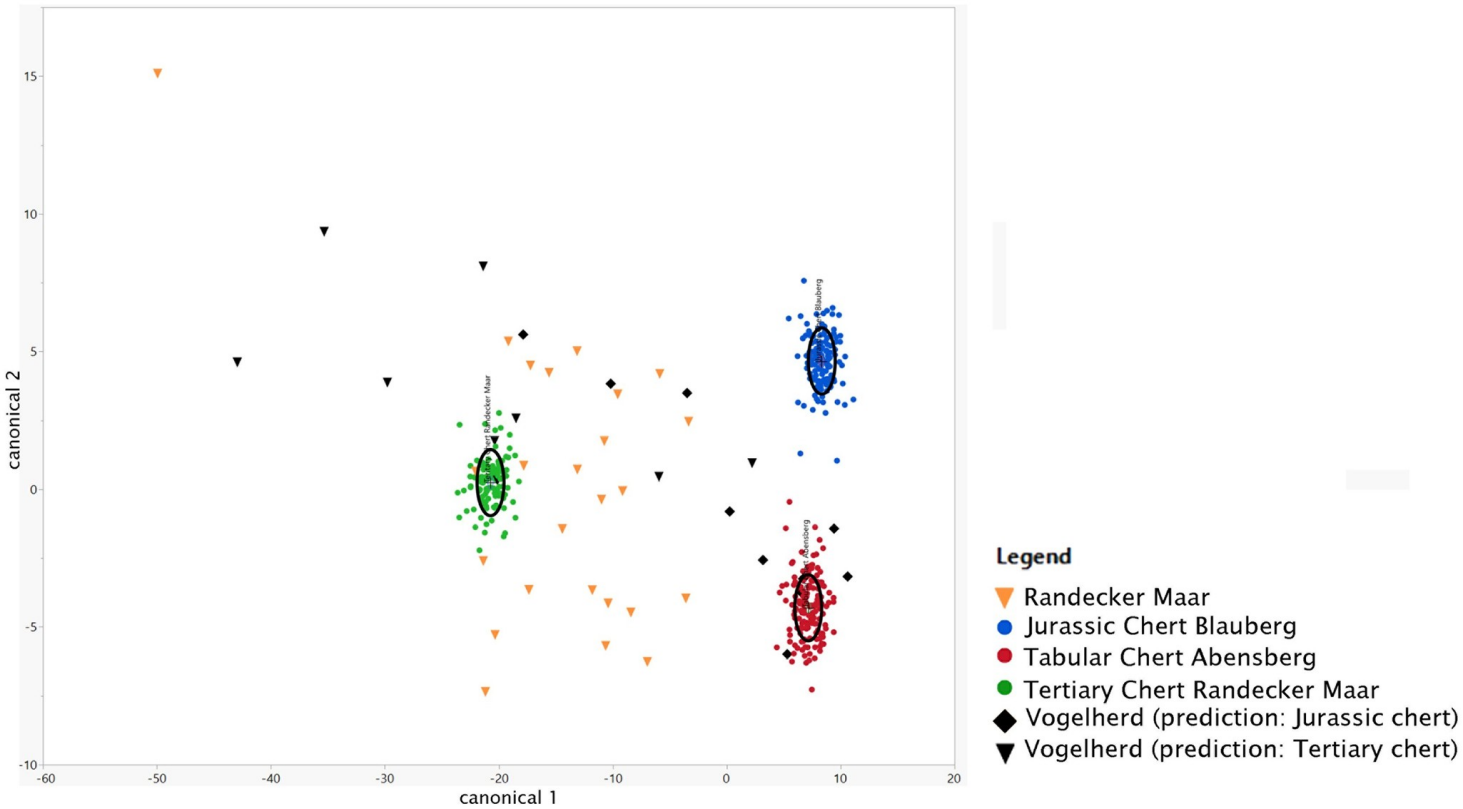
Canonical plot of the LDA. The three groups are coloured in green, blue and red. Archaeological samples are black and orange and were not predefined to a group. The black ellipses encompass 50% of the samples of each group.

The LDA plot (Fig 6) clearly shows that our geological references can be distinguished from each other. For the geological samples, this is also confirmed by the results of the discriminant scores for the geological samples (Table 2). Only four of the 402 geological samples are not correctly classified by LDA. For the archaeological pieces the predicted group membership matches the prediction of the macroscopic analysis in most of the cases. Only five artefacts predicted to be Tertiary chert by the macroscopic analysis are not assigned to the Tertiary chert group by the LDA (Table 3, S1 Table). The archaeological samples from Vogelherd predicted to be Jurassic chert by the macroscopic analysis are all identified as Jurassic chert by the LDA.

**Table 2:** LDA predictions (discriminant-scores) of the geological samples.

Predicted	Observed, LDA	Observed, LDA	Observed, LDA	Accuracy
	Jurassic Chert	Tabular Chert	Tertiary Chert	
Jurassic Chert (BB)	138			100%
Tabular Chert (AA)	4	151		97.40%
Tertiary Chert (RM)			109	100%

**Table 3:** Summary of the predictions of the macroscopic analysis and the predictions of the LDA.

site, layer, age/ raw material identification	Macroscopic Identification	Macroscopic Identification	Observed, LDA	Observed, LDA	Observed, LDA
	Jurassic Chert	Tertiary Chert	Tabular Chert Abensberg	Jurassic Chert Blauberg	Tertiary chert
Vogelherd layer V, Aurignacian	9	1	5	4	1
Vogelherd layer IV, Aurignacian	1	5		1	5
Vogelherd backdirt, mixed	-	3	1	1	1
Randecker Maar, Magdalenian	-	25	2	1	22
Total	10	34	8	7	31

#### Linear discriminant analysis (LDA)—Accuracy tests

To test the accuracy of group membership of our geological reference samples we randomly selected a subsample of 10%, 20% and 30% of our geological refences and removed the information on their group affiliation. As a second step we then performed a LDA and compared the group membership predicted by the LDA with their actual affiliation. In all three cases (10%, 20% and 30%) at least 92.7% of the group memberships were correctly classified (Table 4). Misclassifications only occur between Jurassic chert of Abensberg Arnhofen and Blaubeuren Blauberg. The highest accuracy of random subsamples could be determined for the Tertiary chert from the Randecker Maar. Here all random subsamples were correctly classified by LDA.

**Table 4:** Summary of the LDA accuracy tests.

random subsample (%)	Percentage of correct group membership prediction (%)			
	Total	Abensberg Arnhofen	Blaubeuren Blauberg	Tertiary chert
10	92.7	86.7	85.7	100
20	92.7	81.3	100	100
30	95.2	91.7	90.5	100

## Discussion

The relatively high accuracy value found by our accuracy tests is a good indicator of the ability to separate the different raw materials. 92.7% to 95.2% of our random subsamples were correctly classified. This strongly suggests that LDA is a valid means of separating the three analyzed raw materials. We observe a big overlap of our results from the LDA and PCA. The PCA plot is the main argument to that most of the macroscopic identifications were correct. Moreover, the correspondence of LDA and PCA demonstrates that infrared spectroscopy, as conducted here, can differentiate the raw materials tested in this study. Thus, IR-spectroscopy can make an important contribution to raw material analysis in Southern Germany, especially since past studies were mostly based on macroscopic analysis [9, 10, 12, 13] and not validated with another method. With this analysis, we present one of the few successful archaeometric chert analyses in Southern Germany (for earlier archaeometric approaches see: [16, 55]).

One of the major challenges for raw material studies applied to the sites in the Swabian Alb region is the differentiation of individual outcrops. The amount of raw nodules tested can be crucial for determining the variability in one outcrop. Therefore, future studies will need to show how much variability there is within outcrops and how that affects the results. This is made more difficult by the low macroscopic variability of the raw material deposits and the relatively high level of variability within deposits. Also, in the case of the Randecker Maar, a clear visual macroscopic identification seems to be difficult, even if there are macroscopic characteristics. The importance of secondary impregnation of the raw materials with iron, which occurs regularly for Jurassic cherts still needs to be studied systematically.

For the five archaeological artefacts previously determined as Tertiary chert from the Randecker Maar by macroscopic criteria, our PCA and LDA predicted them to be Jurassic chert. Whether this is really the case or not may also be validated by future studies using fossil inclusions. Characeae are the fossils typically used for the determination of Tertiary chert from the Randecker Maar [56, 57].

## Conclusions

The current study highlights that macroscopic determinations of raw materials in the context of the southern German Upper Palaeolithic need to be verified. Our middle infrared reflectance spectroscopy analysis suggests that some of the artefacts from Vogelherd come from the Randecker Maar, this partially confirms our initial hypothesis that we formulated based on macroscopic methods. This is an important finding, in the light of the facts that most studies of European raw material provenance rely on similar macroscopic criteria.

In the future, we plan to significantly increase the database for southern German raw materials. This may offer the possibility to work out regional differences between raw material deposits, or even differences between specific individual sources in the Swabian Jura. Infrared spectroscopy may well become a welcome new source of data to better understand the territorial behaviour and mobility of the Palaeolithic inhabitants of the Swabian Jura.

From an analytical point of view, further research is needed to better understand the role of surface alteration of the archaeological pieces and how such factors affect the results of IR spectroscopy.

If we can clarify these issues in the future, we may be able to combine data from chert sources with lithic analysis to achieve a better understanding of the Palaeolithic settlement grids or social-economic organization.

## Supporting information

S1 Fig Block 1 and 2 of the raw material samples of Jurassic chert from *Blaubeuren Blauberg*. Photos by B. Schürch.
(TIF)

S2 Fig Block 1 and 2 of the raw material samples of Jurassic chert from *Abensberg Arnhofen*. Photos by B. Schürch.
(TIF)

S3 Fig Block 1 and 2 of the raw material samples of Tertiary chert from *Randecker Maar*. Photos by B. Schürch.
(TIF)

S1 Table Results/predictions of the LDA for the archaeological samples. (XLSX)
