# Supplementary figures and images for: The origin of chert in the Aurignacian of Vogelherd Cave investigated by infrared spectroscopy
Source: PLoS One. 2022 Aug 17;17(8):e0272988. doi: 10.1371/journal.pone.0272988 (PMC9385009; doi:10.1371/journal.pone.0272988)

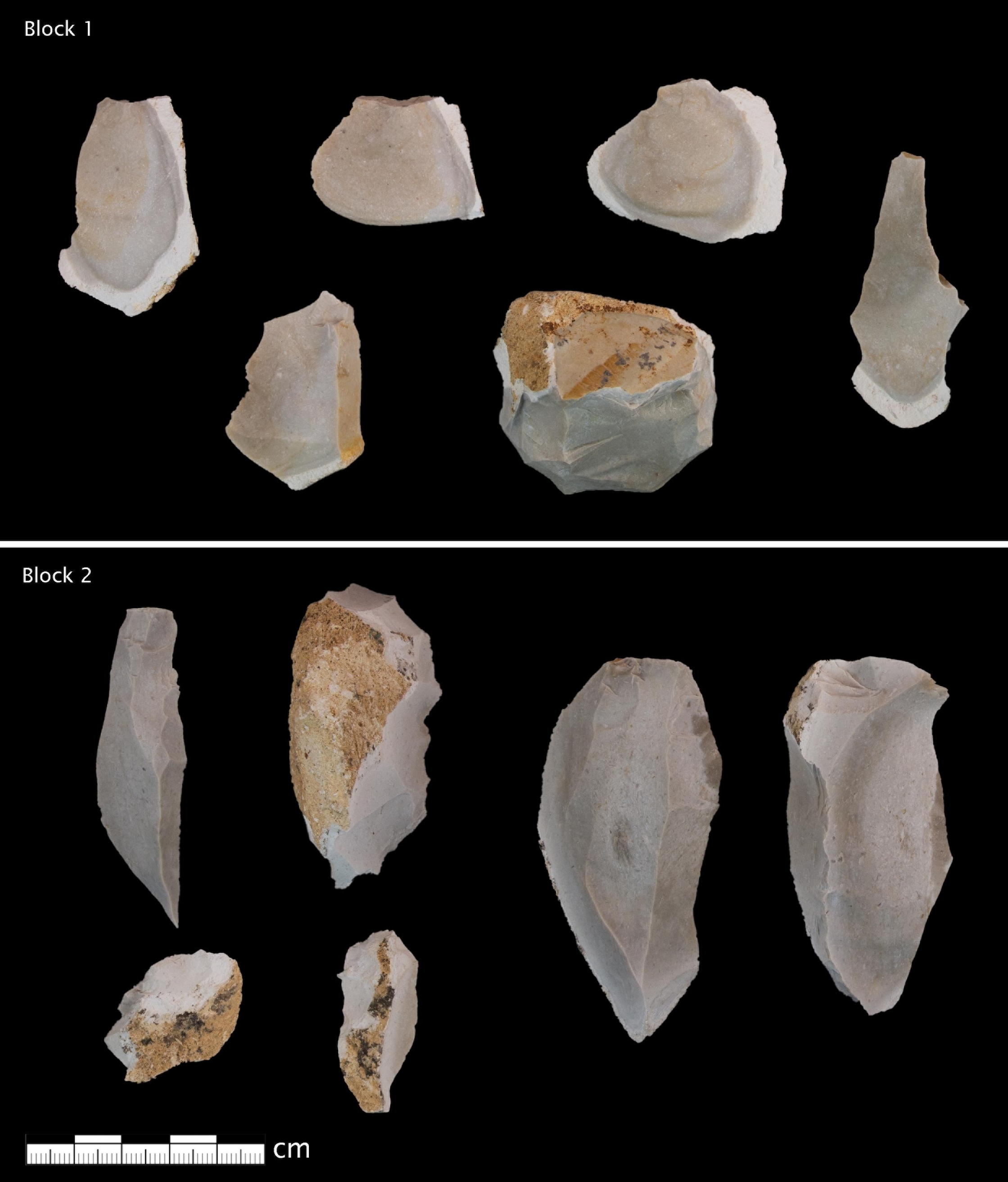

Supplement: S1 Fig — Photos by B. Schürch. (TIF) [file pone.0272988.s001.tif]

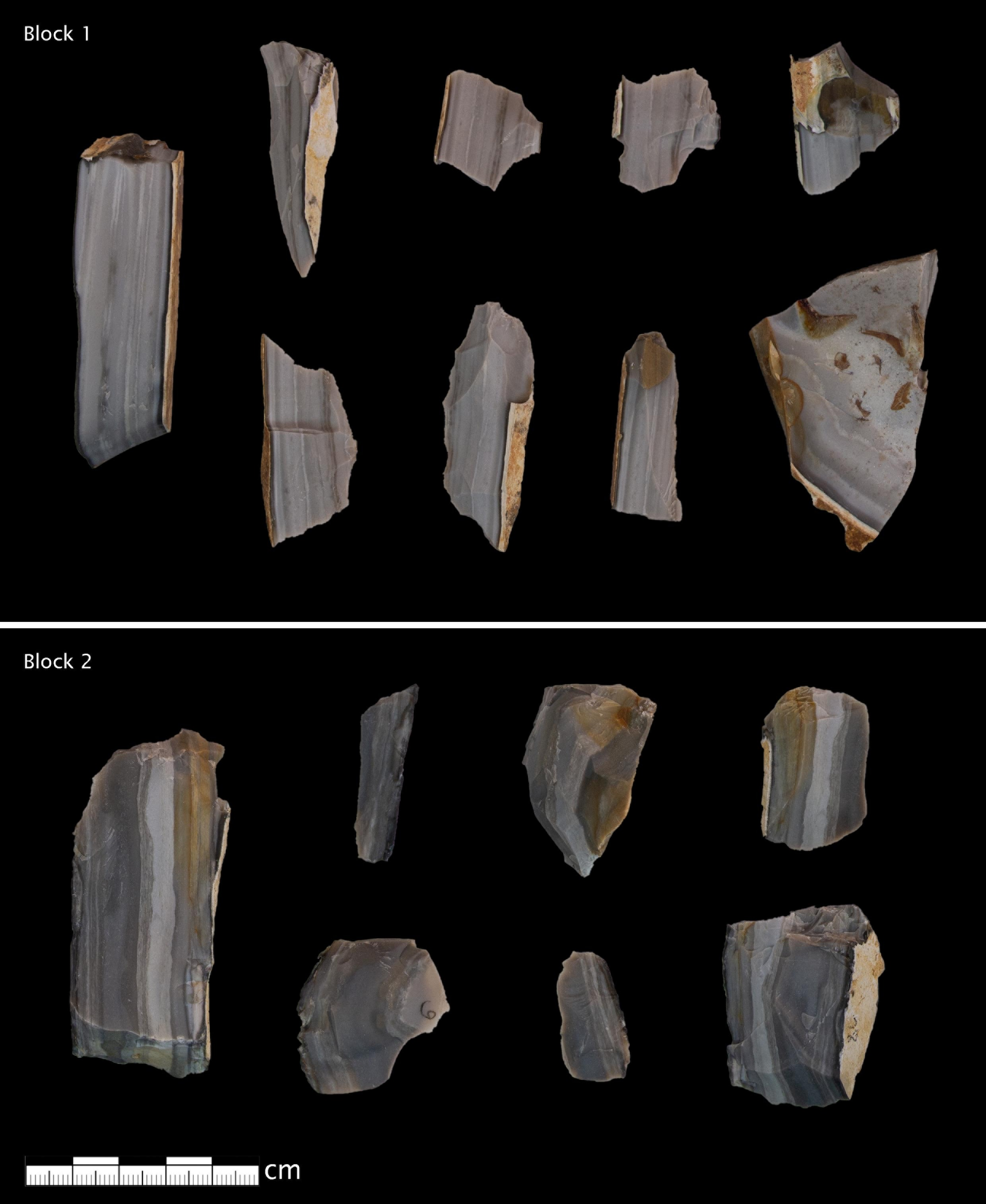

Supplement: S2 Fig — Photos by B. Schürch. (TIF) [file pone.0272988.s002.tif]

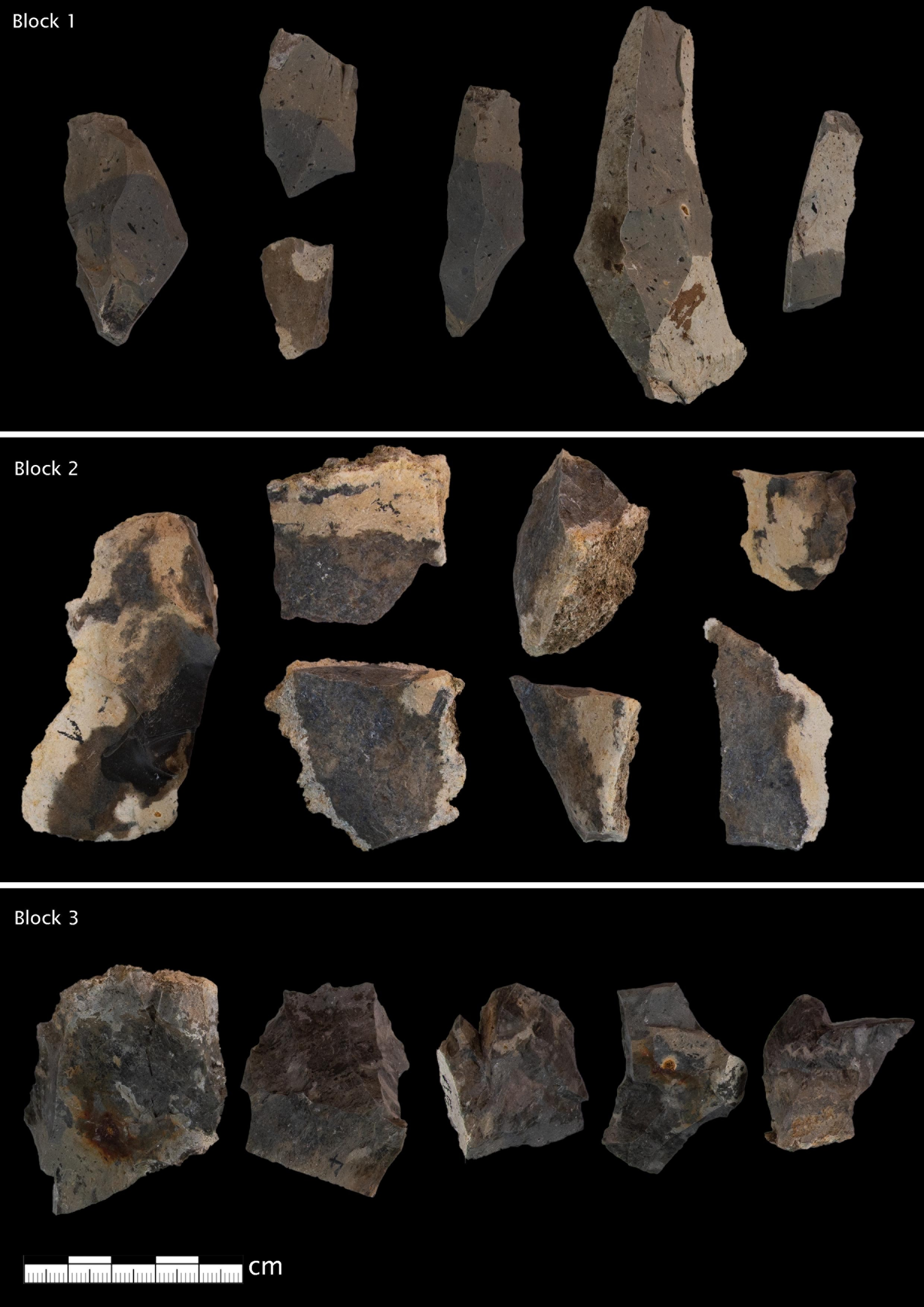

Supplement: S3 Fig — Photos by B. Schürch. (TIF) [file pone.0272988.s003.tif]
